# Supplementary material for: The effect of speech pathology on automatic speaker verification: a large-scale study
Source: Sci Rep. 2023 Nov 22;13:20476. doi: 10.1038/s41598-023-47711-7 (PMC10665418; doi:10.1038/s41598-023-47711-7)
Supplement: Supplementary file 1 — Supplementary Table S1. [file 41598_2023_47711_MOESM1_ESM.pdf]

## Supplementary Information

| | Total num speakers | EER [%] $M = 4$ | Total duration train [hours] | Total duration test [hours] | Age train [years] | Age test [years] |
|---|---|---|---|---|---|---|
| **Adults** | | | | | | |
| dysglossia-dnt-85 | 85 | $2.17 \pm 0.52$ | $3.16 \pm 0.34$ | $0.75 \pm 0.20$ | $58.58 \pm 14.97$ | $57.84 \pm 15.36$ |
| dysarthria-plant-85 | 85 | $1.86 \pm 0.81$ | $1.71 \pm 0.15$ | $0.45 \pm 0.08$ | $60.12 \pm 15.58$ | $60.29 \pm 14.60$ |
| dysphonia-logi-85 | 85 | $1.32 \pm 0.59$ | $1.32 \pm 0.05$ | $0.34 \pm 0.05$ | $58.91 \pm 11.31$ | $59.49 \pm 9.85$ |
| ctrl-plant-A-85 | 85 | $2.04 \pm 0.67$ | $1.28 \pm 0.03$ | $0.32 \pm 0.03$ | $23.78 \pm 15.47$ | $23.98 \pm 14.33$ |
| **Children** | | | | | | |
| CLP-dnt-124 | 124 | $3.67 \pm 0.61$ | $6.79 \pm 0.44$ | $1.77 \pm 0.30$ | $9.48 \pm 3.20$ | $9.31 \pm 3.06$ |
| CLP-plant-124 | 124 | $5.01 \pm 0.69$ | $6.31 \pm 0.10$ | $1.57 \pm 0.10$ | $9.27 \pm 2.54$ | $9.20 \pm 2.40$ |
| ctrl-plant-C-124 | 124 | $3.98 \pm 0.86$ | $5.90 \pm 0.22$ | $1.44 \pm 0.15$ | $11.14 \pm 3.21$ | $11.11 \pm 3.08$ |
| all-children-124 | 124 | $3.14 \pm 0.66$ | $6.03 \pm 0.29$ | $1.54 \pm 0.12$ | $10.54 \pm 3.17$ | $10.49 \pm 3.08$ |
| CLP-dnt-plant-500 | 500 | $1.88 \pm 0.21$ | $26.55 \pm 0.58$ | $6.80 \pm 0.30$ | $10.29 \pm 4.73$ | $10.05 \pm 4.43$ |
| ctrl-plant-C-500 | 500 | $1.93 \pm 0.15$ | $24.08 \pm 0.55$ | $6.03 \pm 0.32$ | $11.72 \pm 3.69$ | $11.70 \pm 3.65$ |
| **Logarithmic effect** | | | | | | |
| all-spk-50 | 50 | $3.75 \pm 1.18$ | $1.98 \pm 0.21$ | $0.56 \pm 0.10$ | $25.50 \pm 22.70$ | $22.06 \pm 18.27$ |
| all-spk-500 | 500 | $1.21 \pm 0.12$ | $20.22 \pm 0.73$ | $4.99 \pm 0.34$ | $24.87 \pm 22.86$ | $24.60 \pm 22.48$ |
| all-spk-1500 | $1,500$ | $0.71 \pm 0.07$ | $60.58 \pm 1.17$ | $15.03 \pm 0.59$ | $25.22 \pm 23.12$ | $25.17 \pm 23.13$ |
| all-spk-3000 | $3,000$ | $0.55 \pm 0.03$ | $120.86 \pm 1.15$ | $30.34 \pm 0.85$ | $25.08 \pm 23.06$ | $25.16 \pm 23.07$ |

**Table S1.** Statistics of the training and test sets of all the experiments. In all the experiments, 20% of the speakers were assigned to test and 80% of the speakers to training. Abbreviations: dysglossia: Patients with dysglossia who underwent prior maxillofacial surgery; dysarthria: Patients diagnosed with dysarthria; dysphonia: Patients with voice disorders; CLP: Children with cleft lip and palate; dnt: Recordings from the "dnt Call 4U Comfort" headset; plant: Recordings via Plantronics Inc. headset; logi: Recordings via Logitech International S.A. headset; ctrl: Control group. The labels "-A" and "-C" respectively indicate adult and children subsets. Numbers appended, such as "-85" in "dysglossia-dnt-85", represent the total speaker count for that experiment. "all-spk" designates experiments combining all dataset speech signals from both adults and children, and both pathological and healthy subjects.
